# Factors Associated with Self-Reported Chronic Diseases of Syrian Refugees in Turkey

**DOI:** 10.5334/aogh.3794

**Published:** 2022-07-27

**Authors:** Mehmet Balcilar, Canan Gulcan

**Affiliations:** 1 Department of Economics, Faculty of Business and Economics, Eastern Mediterranean University, Famagusta, TRNC, via Mersin 10, Turkey; 2 Department of Economics, OSTIM Technical University, Ankara, Turkey

**Keywords:** Syrian refugees, health behaviours, health literacy, chronic diseases, logit regression

## Abstract

**Background:**

Syria’s civil conflict, which began in 2011, led millions of Syrians to migrate to countries all over the world, including Turkey. Considering the fact that war-caused migrations may affect the citizens of the host countries and immigrants from diverse perspectives, It is important to make scientific research on the outcomes of migration after the Syrian civil war.

**Objective:**

This paper investigates the relationship between chronic diseases, such as, cardiac disease, diabetes, and hypertension, and covariates, including socioeconomic status, war-related conditions, risky health behaviours, health services use, and health literacy, using survey data on 7 202 Syrian refugees from 4 068 households living out of camp settlements in Turkey.

**Methods:**

Logistic regressions were employed to examine the relationship between the chronic diseases and covariates, which include socioeconomic status, war-related conditions, risky health behaviours, health services use, and health literacy.

**Findings:**

The results reveal that pre-migration income, health behaviours, such as, tobacco consumption, body mass index, daily activity, health-care use, and health literacy are the most important factors for one or more chronic diseases.

**Conclusion:**

Considering the effects of risk factors on chronic diseases among Syrian refugees, it is critical to take preventive steps for negative outcomes.

## Introduction

The United Nations High Commissioner for Refugees reported that almost 6.6 million Syrians have registered as refugees due to the civil war in Syria [1]. Since 2011, Turkey welcomed 3.74 million Syrians, who have been registered as refugees, and has played a very critical role in saving the lives of millions of Syrians forced to leave their country [2]. As war-triggered immigration deprives people of access to a regular healthcare system, there may be a decline in refugees’ quality of life, living conditions, and health statuses. This requires extensive research about their health statuses to be able to improve the living conditions and health of refugees in Turkey. From this perspective, to determine the health needs and health statuses of refugees living in Turkey, comprehensive research was carried out by World Health Organization in 2018 [3]. Extensive data on topics including demographics, war-related conditions, socioeconomic status (SES), healthcare use, health behaviours, and health literacy factors were obtained, which may be employed for the overall evaluation of the health status of Syrian refugees in Turkey.

There are many factors involved in health outcomes, including the development of chronic disease states. Health literacy is one of the factors to be considered in determining the level of health status. “Health literacy,” a term first introduced in the early 1970s, has been described broadly by the World Health Organization as “the personal characteristics and social resources needed for individuals and communities to access, understand, appraise and use information and services to make decisions about health” [4]. It has been shown that inadequate health literacy influences health negatively [5]. From a broader perspective, limited health literacy was found to be associated with poor health status, limited knowledge about medical care, lack of understanding and use of preventive services, more hospitalizations, and more chronic diseases (e.g., cardiac disease, diabetes, and hypertension) [6,7,8]. Notably, some studies proposed that migrants, the elderly, illiterate individuals, and people with low income tend to have limited health literacy; hence, the majority of individuals in these groups are at risk of poor health status [9,10]. Health behaviours, particularly tobacco use and body mass index (BMI), are also significant indicators of health outcomes. Recent studies reported their direct effects on the development of chronic disease states. Indeed, many metabolic disorders involving hypertension, cardiac disorders, and diabetes have been found to be connected to tobacco use and abnormalities related to BMI values [11,12]. Therefore, determination of the effects of BMI and tobacco use on health outcomes will contribute to investigations of the possible causes of poor health.

It is obvious that maintaining and improving health while simultaneously treating diseases are necessary actions for healthy communities [13]. Studies have shown that people without health insurance have poorer health than those with insurance [14]. It is well known that a lack of health insurance can lead to less access to healthcare, fewer physician visits, a higher likelihood of not receiving medical care for severe health symptoms, and lower use of suggested preventative services [15].

Other factors in the literature considered to be associated with chronic diseases were stated as age, gender, marital status, and socioeconomic factors (e.g., education, income, occupation, and employment). For instance, many disorders appear at older ages, and some diseases can be seen more often in men than women. Furthermore, marital status can have significant effects on one’s lifestyle; therefore, married and unmarried people may have different tendencies in the protection of their health [16]. However, lower educational levels may result in a higher likelihood of developing chronic diseases for both genders [17]. Previous studies declared that education aids in obtaining skills for acquiring social, psychological, and economic assets [18]. The effect of education on health was understood on the basis of the idea that higher education levels lead to healthier lifestyles [19]. Notably, working status is another key element of SES in terms of retaining one’s income and living conditions at a constant level or improving them [20]. In addition, it has been shown that higher SES leads to better health outcomes as it provides access to the health services that one needs [21]. Overall, consideration of certain sociodemographic and socioeconomic factors is necessary in evaluating the health statuses of both individuals and populations.

Based on the factors noted above, it will be of value to conduct an investigation of the health status of Syrian refugees, since little information is available about it and limited studies have been conducted on this topic in Turkey. Therefore, the primary objective of the present study is to assess the effects of war-related conditions, SES, healthcare use, health behaviours, and health literacy on chronic diseases (i.e., cardiac disease, diabetes, and hypertension) among Syrian refugees living outside of refugee camps in Turkey. To the best of our knowledge, this study is the first to examine these specific factors affecting chronic diseases in such a large sample of the Syrian refugee population.

## Materials and methods

### Sample population and questionnaire

In the analyses, the data source allowed us to include over 10 000 Syrian respondents (including children under 15) from 4 068 households living in 15 Turkish provinces. However, in order to achieve the purpose of the present study, a total of 7 202 Syrian refugees over the age of 15 were included. The data were obtained from a survey conducted by the World Health Organization Country Office in Turkey in 2018. The survey was designed to obtain estimates about refugees’ living and health conditions considering local necessities and resources. All survey materials were translated into Arabic, Turkish, and English so that all participants and field staff could understand them. The questions were administered with computer tablets using face-to-face interviews and computer-assisted personal interviewing. Self-reported closed-ended (yes/no) questions were asked to identify the incidence of chronic diseases, such as, cardiac disease, diabetes, and hypertension, among these Syrian refugees. The following were the predictor variables included in the analyses: (1) demographic factors: age, gender, marital status, pre-migration residency, (2) war-related conditions: number of family members who died during the war, houses damaged in Syria during the war, years spent in Turkey, (3) SES factors: education (classified into 5 groups including illiterate, literate but no formal schooling, that is, not received any education, primary-secondary school graduates, high school graduates, and university/postgraduates), pre-migration income, pre-migration working status, (4) health behaviours: tobacco use (categorized into 3 groups: current tobacco users, not current tobacco user but the past user, and not past and current tobacco user), BMI (BMI was calculated using the data based on the height and weight of each respondent), daily activity level (in minutes per day), (5) use of healthcare services following migration (health insurance, healthcare use), and (6) health literacy. The Eastern-Middle Eastern Adult Health Literacy (EMAHL13) screening instrument, designed to measure the health literacy of individuals in Eastern and Middle Eastern regions, was employed to evaluate refugees’ health literacy [22]. Nine of 13 questions from the EMAHL13 screening instrument were combined to provide health literacy scores with answers given according to the standard response scale (i.e., “never=1”, “rarely=2”, “sometimes=3”, “most times=4”, “always=5”). For each respondent, the mean score for all item replies ranged from 9 to 45. In the analyses, the mean score was used to investigate the effect of health literacy level on self-reported chronic diseases of Syrian refugees.

### Statistical analysis

All statistical analyses were conducted using IBM SPSS Statistics 21. To check the associations between the explanatory factors (i.e., demographics, war-related conditions, socioeconomic factors, health behaviours, use of healthcare services, and health literacy) and response variables (i.e., cardiac disease, diabetes, and hypertension), statistical significance tests were applied, including chi-square tests for categorical variables and t-tests for continuous variables. On the other hand, the Wilcoxon test was used to examine differences due to the skewed distribution of scores for some of the continuous variables. Since the response variables were dichotomous (i.e., 1 for the presence of the disease and 0 for the absence of the disease), multiple logistic regression analysis was applied with the aim of determining how 2 or more explanatory variables (categorical or continuous) affected a dichotomous response variable. The general form of the logit of the multiple logistic regression model for two possible outcome levels can be taken as follows: [23]


1 Pr\,\,\left( {Y = 1|x} \right) = ln\left[ {\frac{{\pi (x)}}{{1-\pi (x)}}} \right] = {\beta _{0\, + \,}}{\beta _1}{x_1} \ldots \ldots \ldots \,{\beta _p}{x_p}

\pi (x) = \frac{{{e^{\beta 0 + \beta 1x1 + .. \ldots .\beta pxp}}}}{{1 + {e^{\beta 0 + \beta 1x1 + \ldots \beta pxp}}}}

where Pr (*Y* = 1|*x*) denotes the expected probability that the event is present, *x_1_* ….. *x_p_* represent explanatory variables, *β_0_* is the y-intercept, and *β_1_* …… *β_p_* denote the coefficient estimates. The general equation form for all the models, i.e., cardiac disease, diabetes, and hypertension, to be estimated is presented in equation (1).

## Results

### Descriptive statistics

Table 1 summarizes the data for all of the explanatory and outcome variables used in this research. The mean age of the respondents was 31.5 years and 47.8% of the 7 202 respondents were female while 52.2% were male. The majority of participants were married at a rate of 77%. The mean number of years spent in Turkey after the migration was 3.47 years. The majority of the respondents (60.1%) reported having been located in Aleppo, Syria, before migrating to Turkey. The average number of family members to die during the war was 0.07 and 42.2% of participants stated that their homes in Syria had been completely destroyed.

**Table 1:** Descriptive statistics of the sample population.


EXPLANATORY VARIABLES	N	%	MEAN	STD.DEV.

Demographic Factors:				

Age	7202		31.5	10.978

Gender	7202			

female		47.8	0.52	0.50

male		52.2		

Marital Status	7202			

married		77.0	0.41	0.769

single		17.5		

separated/divorced/widowed		5.5		

Pre-migration residency	6964			

Aleppo		60.1	0.38	0.485

other		36.6		

War-related conditions:	6964			

Damage to the house in Syria		42.2		

completely destroyed		16.0	1.33	1.397

very damaged		8.6		

less damaged		24.0		

I do not know		6.0		

undamaged				

Family member died during the war	6964		0.07	0.370

Years in Turkey	6871		3.47	1.397

SES factors:				

Education	7202			

illiterate		21.9	1.60	.994

literate but no formal schooling		9.0		

primary/secondary school		58.4		

high school		8.2		

university/post-grad.		2.4		

Income in Syria (in log)	6146		8.47	1.962

Working status in Syria	7188		0.37	0.482

working		36.8		

not working		63.0		

Health Risk Behaviours:				

Tobacco use	6964			

current tobacco user		13.4	1.71	0.695

not current tobacco user but the past user		1.4		

not past and current tobacco user		81.9		

BMI	6794			

healthy weight		58.2	2.20	1.044

underweight		2.7		

overweight		28.4		

obese		5.1		

Daily activity	6964		16.85	9.329

Health Services Use:				

Health insurance	6964			

yes		5.5	0.06	0.232

no		91.2		

Health-care services use	6964			

yes		65.1	0.67	0.469

no		31.6		

Health Literacy	6964		18.12	9.591


Among SES factors, the mean pre-migration income of the refugees participating in the survey was 8.47 (in log). A majority of the participants were primary/secondary school graduates (58.4%). As can be seen in Table 1, a majority of respondents identified themselves as not working in Syria (63.0%). Of the respondents, 58.2% did not use tobacco in the past or at present (81.9%) and had a healthy weight. Although the use of healthcare services among these Syrian refugees was found to be considerably high (65.1%), a majority reported having no health insurance (91.2%). They also had low levels of health literacy, with an average score of 18.12.

### Empirical results of factors affecting chronic diseases

Tables 2, 3 and 4 summarize the logistic regression estimates for the associations among demographic factors, war-related conditions, SES, health behaviours, use of healthcare services, health literacy, and chronic diseases (i.e., cardiac disease, diabetes, and hypertension). The results are presented in these tables with the coefficient estimates (*β*), odds ratios (ORs), and confidence intervals (95%) for each variable.

**Table 2:** Multiple logistic regression estimates.


*DEPENDENT VARIABLE (CARDIAC DISEASE^a^)*	β	PROB.	OR (CI 95%)

***Demographic Factors***			

Age	.080***	.000	1.083 (1.065–1.102)

Gender^b^			

female	.231	.488	1.260 (0.656–2.420)

Marital Status^c^			

married	–.798*	.042	0.450 (0.209–0.972)

separated/divorced/widowed	–.460	.375	0.631 (0.229–1.743)

Pre-migration residency^d^			

Aleppo	–.098	.670	0.907 (0.578–1.422)

***War–related conditions***			

Number of family members who died during the war	.229	.191	1.257 (0.892–1.773)

Damage to the house in Syria^e^			

completely destroyed	–.075	.880	0.927 (0.348–2.475)

very damaged	.285	.582	1.330 (0.482–3.674)

less damaged	.548	.310	1.729 (0.600–4.982)

I don’t know	.282	.582	1.326 (0.486–3.617)

Years spent in Turkey	.071	.369	1.073 (0.920–1.252)

***Socio-economic status***			

Education^f^			

ılleterate	–.055	.934	0.946 (0.258–3.478)

literate	–.057	.941	0.945 (0.213–4.200)

primary/secondary school	–.326	.614	0.722 (0.204–2.556)

high school	–.232	.749	0.793 (0.190–3.299)

Pre-migration income (in log)	.074	.243	1.076 (0.951–1.218)

Pre-migration working status^g^			

not working	–.304	.321	0.738 (0.405–1.345)

***Health risk behaviours***			

Tobacco use^h^			

current user	.757**	.002	2.132 (1.316–3.455)

not current user but the past user	.045	.944	1.046 (0.296–3.699)

BMI^i^			

obese	1.217***	.000	3.378 (1.799–6.345)

overweight	.581*	.013	1.788 (1.129–2.833)

underweight	.397	.600	1.487 (0.338–6.534)

Daily activity (minutes)	–.002	.872	0.998 (0.974–1.022)

***Health services***			

Health insurance^j^			

no	–.335	.396	0.715 (0.330–1.551)

Health-care use^k^			

no	–.976**	.001	0.377 (0.211–0.674)

***Health literacy***	–.028	.052	0.972 (0.945–1.000)

Pseudo R-Square	.203		


*Note*: Reference categories are (a) no; (b) male; (c) single; (d) other; (e) undamaged; (f) university/post graduate; (g) working; (h) not past and current tobacco user; (i) healthy weight; (j) yes; (k) yes. *** p < 0.001; ** p < 0.01; and * p < 0.05.

**Table 3:** Multiple logistic regression estimates.


*DEPENDENT VARIABLE (DIABETES^a^)*	β	PROB.	OR (CI 95%)

***Demographic Factors***			

Age	.085***	.000	1.089 (1.070–1.107)

Gender^b^			

female	.129	.688	1.138 (0.607–2.132)

Marital Status^c^			

married	–1.298***	.000	0.273 (0.139–0.537)

separated/divorced/widowed	–1.010**	.035	0.364 (0.143–0.930)

Pre-migration residency^d^			

Aleppo	–.110	.622	0.896 (0.580–1.386)

***War–related conditions***			

Number of family members who died during the war	.041	.592	1.042 (0.896–1.212)

Damage to the house in Syria^e^			

completely destroyed	–.182	.695	0.833 (0.335–2.072)

very damaged	.519	.273	1.680 (0.664–4.251)

less damaged	–.187	.733	0.829 (0.283–2.429)

ı don’t know	–.156	.752	0.856 (0.325–2.249)

Years in Turkey	.041	.592	1.042 (0.896–1.212)

***Socio–economic status***			

Education^f^			

ılleterate	–.146	.829	0.864 (0.231–3.240)

literate	–.763	.392	0.466 (0.081–2.674)

primary/secondary school	–.101	.877	0.904 (0.253–3.232)

high school	.302	.668	1.352 (0.341–5.357)

Pre–migration income (in log)	.135*	.032	1.144 (1.012–1.294)

Pre–migration working status^g^			

not working	–.159	.589	0.853 (0.480–1.517)

***Health risk behaviours***			

Tobacco use^h^			

current user	.228	.400	1.256 (0.738–2.138)

not current user but the past user	1.072**	.017	2.921 (1.212–7.040)

BMI^i^			

obese	1.349***	.000	3.855 (2.088–7.117)

overweight	.558*	.016	1.747 (1.108–2.754)

underweight	.242	.750	1.274 (0.287–5.660)

Daily activity (minutes)	–.024*	.044	0.976 (0.953–0.999)

***Health services***			

Health insurance^j^			

no	1.281*	.040	3.600 (1.057–12.26)

Health–care use^k^			

no	–.977**	.001	0.377 (0.210–0.675)

Health literacy	–.034*	.014	0.966 (0.940–0.993)

Pseudo R–Square	.221		


*Note*: Reference categories are (a) no; (b) male; (c) single; (d) other; (e) undamaged; (f) university/post graduate; (g) working; (h) not past and current tobacco user ; (i) healthy weight; (j) yes; (k) yes. *** p < 0.001; ** p < 0.01; and * p < 0.05.

**Table 4:** Multiple logistic regression estimates.


*DEPENDENT VARIABLE (HYPERTENSION^a^)*	β	PROB.	OR (CI 95%)

***Demographic Factors***			

Age	.069***	.000	1.072 (1.057–1.087)

Gender^b^			

female	.600*	.021	1.822 (1.096–3.029)

Marital Status^c^			

married	–.611*	.037	0.543 (0.306–0.963)

separated/divorced/widowed	–.658	.112	0.518 (0.230–1.165)

Pre–migration residency^d^			

Aleppo	–.073	.684	0.929 (0.653–1.323)

***War–related conditions***			

Number of family members who died during the war	.391**	.001	1.479 (1.173–1.865)

Damage to the house in Syria^e^			

completely destroyed	.101	.797	1.106 (0.512–2.393)

very damaged	.592	.143	1.808 (0.819–3.994)

less damaged	.336	.448	1.400 (0.587–3.338)

I don’t know	.190	.645	1.209 (0.539–2.709)

Years in Turkey	.029	.624	1.030 (0.915–1.159)

***Socio–economic status***			

Education^f^			

ılleterate	–.195	.706	0.823 (0.298–2.267)

literate	–.855	.191	0.425 (0.118–1.532)

primary/secondary school	–.261	.599	0.770 (0.291–2.039)

high school	–.037	.946	0.964 (0.329–2.825)

Pre–migration income (in log)	.079	.102	1.083 (0.984–1.191)

Pre–migration working status^g^			

not working	–.107	.671	0.899 (0.549–1.471)

***Health risk behaviours***			

Tobacco use^h^			

current user	.370	.086	1.447 (0.949–2.206)

not current user but the past user	.680	.122	1.974 (0.834–4.674)

BMI^i^			

obese	1.240***	.000	3.456 (2.132–5.604)

overweight	.539**	.003	1.714 (1.197–2.455)

underweight	.933*	.039	2.543 (1.047–6.180)

Daily activity (minutes)	.001	.925	1.001 (0.982–1.020)

***Health services***			

Health insurance^j^			

no	–.226	.464	0.798 (0.435–1.462)

Health–care use^k^			

no	–.784***	.000	0.456 0(.295–.0705)

***Health literacy***	–.024*	.023	0.977 0(.957–0.997)

Pseudo R–Square	0.153		


*Note*: Reference categories are (a) no; (b) male; (c) single; (d) other; (e) undamaged; (f) university/post graduate; (g) working; (h) not past and current tobacco user; (i) healthy weight; (j) yes; (k) yes. *** p < 0.001; ** p < 0.01; and * p < 0.05.

As shown in Tables 2, 3 and 4, the results reveal significant and positive coefficients between age and the respondents’ chronic diseases. Older respondents are more likely to experience chronic diseases. While gender is significantly associated with hypertension, it does not contribute significantly to cardiac disease or diabetes. Accordingly, female respondents are more likely to have been diagnosed with hypertension than male respondents (OR = 1.82, 95% CI= 1.096 to 3.029). Marital status, on the other hand, is seen to be a statistically significant factor for all 3 chronic diseases, and the odds of being diagnosed with cardiac disease, diabetes, and hypertension decrease by respective factors of 0.45, 0.27, and 0.54 for married refugees. Refugees who are separated, divorced, or widowed are less likely than never-married respondents to have been diagnosed with diabetes by a factor of 0.364.

Respondents whose family members died during the war are more likely to have hypertension, but this was not found to be a risk factor for cardiac disease or diabetes. In other words, those who lost a family member during the war are more likely to have been diagnosed with hypertension by a factor of 1.47 (95% CI = 0.892 to 1.773).

Pre-migration incomes of the refugees were found to be risk factors for experiencing diabetes. As shown in Table 3, pre-migration income has a positive and significant coefficient for diabetes among these refugees (OR = 1.114, 95% CI = 1.012 to 1.294), but it does not contribute significantly to cardiac disease or hypertension.

The findings further indicate that tobacco use is a significant risk factor for cardiac disease and diabetes. The probability of being diagnosed with cardiac disease is 2.13 times higher for tobacco users. Similarly, the odds of having been diagnosed with diabetes are 2.92 times higher for those who are former tobacco users compared to those who have never used tobacco. Notably, it can also be seen in Tables 2, 3 and 4 that BMI is a key parameter for all chronic diseases. Those who are obese and overweight are more likely to have cardiac disease, diabetes, and hypertension. It is also seen that those who are underweight are 2.5 times more likely to have been diagnosed with hypertension compared to those of healthy weight. Among health factors, daily activity is negatively associated with diabetes. With respect to the results shown in Table 3, the odds of being diagnosed with diabetes decrease by a factor of 0.976 as daily activity levels increase.

Another determinant proven to be a highly significant factor for all investigated chronic diseases is the use of healthcare services, shown to be negatively related to cardiac disease, diabetes, and hypertension. In other words, those who do not use healthcare services are less likely to be diagnosed with these chronic diseases. Health insurance was only found to be related to diabetes. Accordingly, participants who have no health insurance are 3.6 times more likely to be diagnosed with diabetes than those who have it (95% CI = 1.057 to 12.26).

From the parameters shown in Tables 3 and 4, it is clear that the health literacy levels of these Syrian refugees are significantly associated with diabetes and hypertension. The results obtained from the analyses reveal that those participants with higher health literacy are less likely to have been diagnosed with diabetes or hypertension than those with lower health literacy. Accordingly, as health literacy increases, the odds of being diagnosed with diabetes decrease by a factor of 0.966 (95% CI = 0.940 to 0.993), and the odds of being diagnosed with hypertension decrease by a factor of 0.977 (95% CI = 0.957 to 0.997). However, we found no effects of years spent in Turkey, pre-migration residency, damage to homes in Syria, education, or the working status of these Syrian refugees on any of the considered chronic diseases.

## Discussion

Our findings may be interpreted in a variety of ways. First, we discovered that age is a significant risk factor for all chronic diseases analyzed in this study. This observation is in parallel with data in the literature indicating a higher risk of chronic diseases among elderly populations [24,25]. Female respondents, as shown in Table 4, are also more likely to have been diagnosed with hypertension compared to male participants. The odds of being diagnosed with hypertension among women are 1.8 times higher than that among men. This is in contrast to the data in the literature since men generally have a slightly higher tendency of developing hypertension with age compared to women [26]. Moreover, we observed that married refugees were less likely to have been diagnosed with cardiac disease, diabetes, or hypertension compared to single participants. In the past few decades, researchers have grown more interested in the associations between marital status and several health outcomes, including chronic diseases. Studies have shown that marital status (i.e., married, single, separated, divorced, or widowed) is linked to health-related outcomes, including death from all causes [27]. It has also been reported that being married is linked to a lower risk of heart disease and stronger overall health status [27]. Indeed, previous studies indicated that married people have lower rates of diabetes in comparison to unmarried people [28]. The reasons for this lower incidence of diabetes among married or partnered individuals are suggested to be the positive effects of partnership on the individual’s health behaviours and SES [29]. Therefore, our findings are compatible with the data available in the literature. Hypertension is a major cause of morbidity and mortality, as well as a major risk factor for cardiovascular diseases [30]. The link between marital status and hypertension has been documented in numerous studies [31]. Since marital status may affect one’s lifestyle, married and single people may have different tendencies in the protection of their health [15].

We observed a higher probability of having hypertension among refugees who had experienced the death of a family member during the war. Previous research focused on health status among Syrians yielded similar results as both female and male refugees who had experienced the death of a family member during the war had higher rates of hypertension [32].

Notably, pre-migration income has been shown to be associated with diabetes, implying that as income increases, the odds of being diagnosed with diabetes also increase. It is well known that diabetes, one of the leading causes of mortality in the world, causes serious damage to the heart, blood vessels, eyes, kidneys, and nerves over time [33]. Obesity, lifestyle, diet, ethnicity, recent immigration, and income have all been identified as risk factors for diabetes [34]. Obesity, as one of these risk factors, was previously observed to be more prevalent in groups with higher SES in studies conducted in developing countries [35]. Although income may have a positive effect on an individual’s health, it is also possible that it might yield opposite results for health, particularly considering its effects on obesity, lifestyle, and diet. Therefore, the general effects of income on the development of diabetes cannot be denied.

Additionally, we found that current tobacco users were more likely to have been diagnosed with cardiac disease, while past tobacco users were more likely to have been diagnosed with diabetes. Tobacco use has been shown to have a direct relationship with the development of several diseases, including chronic diseases [36]. The direct relationship between tobacco use and cardiac disease development is well characterized and our findings thus correlate well with the data in the literature [37]. Additionally, the odds of having cardiac disease, diabetes, and hypertension among obese and overweight refugees were seen to be considerably high compared to refugees of healthy weight. Since both obesity and overweight can trigger the development of the diseases considered here, our results are in accordance with the information obtained to date on this topic [38]. These results reveal that daily activity, as an important health behaviour, helps to decrease the risk of a diagnosis of diabetes. It is known that physical activity in diabetic patients improves glycaemic control and lowers the risk of mortality [39].

It is well recognized that lack of health insurance may lead to poorer health status as it can prevent access to basic healthcare services [40]. In this study, however, the results regarding the association between health insurance and the prevalence of chronic diseases showed that only diabetes is positively associated with health insurance. In other words, the odds of being diagnosed with diabetes are higher among refugees with no health insurance. As shown in Table 1, the frequency of accessing healthcare services is considerably high while the frequency of having health insurance is low among Syrian refugees living in Turkey. Although the majority of the refugees participating in this survey declared no health insurance, they were provided free access to healthcare services and treatment [41]. This allowed them to obtain healthcare services without possessing legal health insurance.

It was observed in the present research that health literacy was significantly associated with diagnoses of diabetes and hypertension but not the cardiac disease. Accordingly, those who have higher levels of health literacy are less likely to be diagnosed with diabetes or hypertension. Poor health literacy is known to be related to the development of chronic diseases with comorbid conditions [42,43]. Accordingly, the association found here between poor health literacy and the presence of diabetes or hypertension is in keeping with previous findings [42,43].

In previous studies, also considering non-communicable disease presence in Syrian refugees, some well-established risk factors (e.g., poor nutrition, tobacco consumption) have been determined [44]. Furthermore, 9% to 50% of the refugees hosted were diagnosed at least with one chronic disease including diabetes, hypertension, abdominal or cardiovascular disorders [45]. From this perspective, the results of this study are not only in parallel with the previous findings related to chronic diseases among refugees but also provide additional information on the chronic diseases and associated factors.

## Conclusion

The civil war that began in Syria in 2011 triggered the migration of millions of Syrians to neighbouring countries, including Turkey. Turkey has become a host country for about 3.74 million Syrian refugees today and several surveys to collect data on the outcomes of this massive migration have accordingly been conducted, particularly focusing on social, psychological, health, and economic aspects. The health status of refugees also needs to be investigated as the conditions under which refugees live might cause serious health risks. To the best of our knowledge, the present study, based on a survey performed by the World Health Organization Country Office in Turkey, is the first extensive refugee-based study to examine the risks of chronic diseases among Syrian refugees living in Turkey.

In this study, a number of factors including demographic factors, war-related conditions, SES, use of healthcare services, health behaviours, and health literacy were examined in relation to chronic diseases among Syrian refugees living in Turkey. In parallel to previous findings on chronic diseases in any population, it was observed that age, marital status, pre-migration income, tobacco use, BMI, daily activity levels, health insurance, use of healthcare services, and health literacy were significantly associated with the presence of one or more chronic diseases among Syrian refugees.

Chronic diseases generally develop over long-term periods; therefore, it may not be possible to easily associate the chronic diseases diagnosed in Syrian refugees with the start of the Syrian civil war or conditions including migration following the beginning of the war. In other words, it is possible that many of the chronic diseases now present in Syrian refugees were already outcomes of the living conditions of these Syrians from young ages. With this in mind, it appears to be a good starting point to identify patients with chronic diseases among Syrian refugees in Turkey, as has been done in the present work. These patients should be accepted as regular citizens of Turkey and their treatment options should be evaluated accordingly. For instance, high BMI and tobacco use are important indicators of the development of chronic diseases and so preventive and treatment strategies might be designed on the basis of education-based activities for Syrian refugees with the aim of teaching them the dangerous adverse effects of tobacco use and unhealthy diets. Such education programs should also include activities on health literacy since higher levels of health literacy are acknowledged to reduce the risks of the development of many disease states including chronic diseases.

One of the important aspects of Turkey’s help in hosting Syrian refugees is its support in providing free healthcare services for this population. This is an important advantage for progressive health programs designed to both lower the levels of chronic diseases observed in this population and make Syrian refugees more conscious of the development of chronic diseases as a result of unhealthy lifestyles.

There may be some possible limitations that could be addressed in future research studies. The primary limitation of this study is that it is unknown whether Syrian refugees had chronic diseases (i.e., cardiac disease, diabetes, and hypertension) before or after migration to Turkey.
